# Iopamidol Abatement from Waters: A Rigorous Approach to Determine Physicochemical Parameters Needed to Scale Up from Batch to Continuous Operation

**DOI:** 10.1021/acs.langmuir.3c02992

**Published:** 2023-12-12

**Authors:** Rosanna Paparo, Michele Emanuele Fortunato, Gianfranco Carotenuto, Fulvio Uggeri, Luigi Nicolais, Martino Di Serio, Marco Trifuoggi, Vincenzo Russo

**Affiliations:** † Chemical Sciences Department, University of Naples Federico II, IT-80126 Naples, Italy; ‡ CeSMA—Centre of Meteorologic and Avanced Thecnology Services, University of Naples Federico II, corso N. Protopisani 70, 80146 Naples, Italy; § Institute for Polymers, Composites, and Biomaterials, National Research Council, SS Napoli/Portici, Piazzale Enrico Fermi 1, 80055 Portici, Italy; ∥ Bracco SpA, via Caduti di Marcinelle, 13, 20134 Milano, Italy; ⊥ Materias Srl, corso N. Protopisani 70, 80146 Naples, Italy

## Abstract

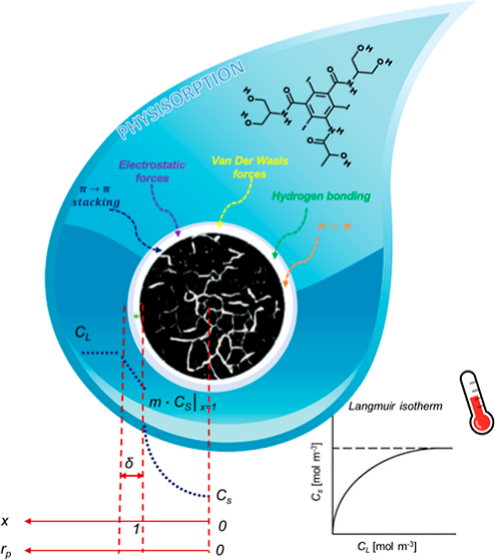

The abatement of iopamidol (IPM), an X-ray iodinated contrast agent, in aqueous solution using powdered activated carbon (PAC) as a sorbent was investigated in the present work. The material was characterized by various analytical techniques such as thermogravimetric analysis, scanning electron microscopy, transmission electron microscopy, Brunauer–Emmett–Teller analysis, dynamic light scattering, and zeta potential measurements. Both thermodynamic and kinetic experiments were conducted in a batch apparatus, and the effects of the initial concentration of IPM, the temperature, and the adsorbent bulk density on the adsorption kinetics were investigated. The adsorption isotherms were interpreted well using the Langmuir model. Moreover, it was demonstrated that IPM adsorption on PAC is spontaneous and exothermic (Δ*H*^0^ = −27 kJ mol^–1^). The adsorption kinetic data were described using a dynamic intraparticle model for fluid–solid adsorption kinetics (ADIM) allowing determination of a surface activation energy *E*_s_ = 6 ± 1 kJ mol^–1^. Comparing the experimental results and the model predictions, a good model fit was obtained.

## 1 Introduction

The paramount use of personal care products (PPCPs) and pharmaceuticals coupled with the limitations of existing wastewater treatment processes have led to research on how to improve modern technique avoiding PPCP diffusion in the natural environment.^1−3^ Organic micropollutants may cause severe ecosystem and health problems even at very low concentrations due to their recalcitrant nature.^4,5^ Iodinated X-ray contrast media (ICMs), a class of 2,4,6-triiodine benzoic acid derivatives, are among the most used injectables in radiology today.^5−8^ Nonionic, iodinated X-ray contrast mediums are highly soluble and inert drugs, thanks to the symmetric triiodobenzene ring structures, leading to their high chemical stability,^6^ which makes it complicated for them to be totally removed by conventional water treatment methods.^7^

The X-ray contrast agents are considered as emerging contaminants and due to a lack of regulatory requirements, they are not routinely monitored and their impact on organisms is not yet totally understood.^8^ Their presence was not reported in surface waters^9,10^ and to a minor extent in groundwater.^11,12^ ICMs are subministrated at high concentrations (up to 200 g/day) and eliminated primarily in their nonmetabolized form in urine and feces. Iopamidol (IPM) is one of the most widespread contrast media characterized by high water solubility and low toxicity, which means that it can be safely injected intravenously at very high doses (up to 400 mg/mL).^13^ However, IPM was detected at concentrations as high as 2.7 mg/L in raw waters,^14^ 16 mg/L in effluents of wastewater treatment plants (WWTPs),^15^ and 1.9 mg/L in sources of drinking water.^16,17^ IPM is a nonionic X-ray contrast medium characterized by a triiodinated benzene structure containing amide and hydroxyl functionalities. Since not much is known about its destiny and long-term consequences, there is a risk connected to its diffusion in the environment^17,18^ At neutral pH it is uncharged with a high hydrophilic property displaying high aqueous solubility, resulting in the fact that it is difficult to it take away from water and wastewater. The physicochemical properties of IPM are shown in Table 1.

**Table 1:**
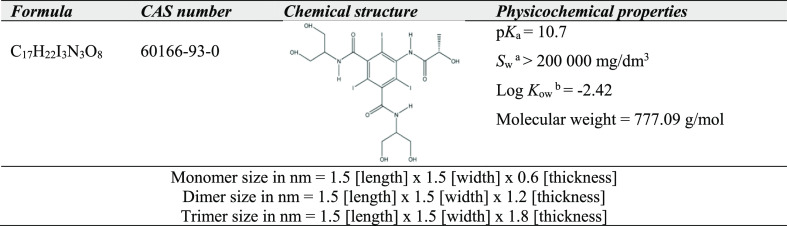
IPM Physicochemical Properties^17,18^

a *S*_w_: solubility in water.

b *K*_ow_: octanol–water partition coefficient.

Several studies found that IPM can be efficiently removed from waters using CuO/PMSa (peroxymonosulfate),^16^ Fe(VI) oxidation,^19^ either chlorine^20^ or UV–UV/chlorine treatment,^21^ photocatalytic oxidation,^22,23^ anaerobic transformation,^24^ etc. Nowadays, the conventional wastewater treatment methods (e.g., coagulation and sedimentation) are not able to treat IPM efficiently^25,26^ and the result is the release of IPM in the aquatic environment at the level of μg/L, proving dangerous for human health and the natural environment. Furthermore, IPM is a potential source of detrimental iodinated byproducts such as iodinated trihalomethanes formed during treatment with chlorine, which are more toxic than iopamidol (IPM) itself.^27^ Adsorption, compared to other techniques, is the most widely used to remove organic pollutants from water because of high efficiency, low regeneration cost, and easy operations, without production of dangerous byproducts.^9,10^ Nowadays, the process of adsorption is still recognized as one of the most common treatment methods to purify and recycle effluents containing inorganic or organic molecules.^28,29^ For this reason, many scientists and engineers are searching for more nonconventional^30^ adsorbent materials [carbon nanotubes,^31^ graphene,^32^ activated carbon (AC), metal-oxide based nanomaterials, polymer-based nanomaterials,^33−35^ polysaccharides,^36,37^ composites of carbon,^38^ and metal-oxide]. Among them, graphene is a multifunctional product that can be used as an adsorbent for removal of oils, organic solvents, and dyes^39,40^ from contaminated water as well as an electrode material for supercapacitors.^41^

AC is a versatile adsorbent due to its large specific surface area, high pore volume, high adsorption capacity, availability of many variants, high purity, high chemical, and thermal stability.^42^ However, as-prepared ACs are usually nonselective and rather expensive,^43^ even if easily regenerable, either by using dedicated solvents or at high temperature. In fact, AC is used to purify, decolorize, deodorize, separate, and concentrate constituents from gases or liquid solutions. For this reason, AC is used in different sectors such as pharmaceuticals, food, dye, petroleum, chemical nuclear, vacuum industries and automobiles, as well as for the treatment of drinking water and urban and industrial wastewater.^29^ A summary of the most recent efforts made in the adsorptive removal of IPM using AC is reported in Table 2, highlighting the implemented technology.

**Table 2:** Some Previously Published Works on the Adsorptive Removal of IPM Using AC^a^

type of AC	matrix tested	implemented technology	removal efficiency [%]	*q*_e_ [mg/g]	reference
commercial powdered AC (PAC)	WWTP Schonerlinde effluent sample	horizontal lab shaker			(44)
granular AC (GAC)	mixture of 10 pollutants (IPM standard)	fixed-bed filters	fresh GAC = 90, used GAC = 30		(45)
precursor of granules of expanded corkboard	IPM supplied by Hovione	batch system	S800 = 90, CP = 70, VP = 65	S800 = 137, CP = 123, VP = 115.5	(46)
sucrose-derived ACs	IPM supplied by Hovione	batch system	SH800 = 89.6, SC800 = 16, NS = 42.9	SH800 = 806, SC800 = 144, NS = 386	(47)
wood powder AC (WPAC) peat powder AC (PPAC) peat granular AC (PGAC) Coconut powder AC (CPAC)	IPM supplied by Bracco	batch and flow study	PGAC = 25, PPAC = 62, WPAC = 82, CPAC = 95	PGAC = 120, PPAC = 350, WPAC = 450, CPAC = 500	(48)
lab-made carbons compared with commercial AC (CP, VP)	IPM supplied by Hovione	glass vials	CP = 70, VP = 65, S3 = 25	CP = 150, VP = 140, S3 = 110	(17)

a Removal efficiency [%] = [(*C*_0_ – *C*_e_)/*C*_0_] × 100, where *C*_0_ and *C*_e_ are the initial and equilibrium IPM concentrations. *q*_e_ [mg/g]: amount of IPM adsorption at equilibrium, (see eq 1). S800: activation of the solid with steam at 800 °C. SH800: activation of the solid with KOH at 800 °C. SC800: activation of the solid with K_2_CO_3_ at 800 °C. CP, VP, NS: commercial carbons.

Even though several articles have been published until now on IPM adsorption on AC, a dedicated kinetic and thermodynamic investigation is still missing. In particular, the aim of this study is to determine the kinetic and thermodynamic information that will be needed to design an adsorption column working in flow.

## 2 Materials and Methods

### 2.1 Materials

IPM solution was prepared using ISOVUE-370 solution (Bracco Diagnostics, 76% w/v); Iopamidol Pharmaceutical Secondary Standard (Certified Reference Material Sigma-Aldrich), acetonitrile (MW 41.053 g/mol, CAS 75-05-8, Honeywell Chromasolv, for HPLC, for UV, 99.9%), sodium hydroxide (MW 40 g/mol, ≥98%, CAS 1310-73-2, Honeywell, Fluka), and hydrochloric acid 0.1 M (MW 36.46 g/mol, CAS number purchased from 7647-01-0, purchased from TITOLCHIMICA S.p.a.) were used. Activated charcoal (Fluka Chemika 05105, CAS 7440-44-0) was used as the adsorbent material. Deionized water (conductivity ≤ 2 μS/cm) was used to prepare the solutions. All of the materials were used without any further treatment.

### 2.2 Methods

#### 2.2.1 Physical-Chemical Characterization of the Adsorbent

The adsorbent material was fully characterized regarding its morphology and structure by transmission electron microscopy (TEM), dynamic light scattering (DLS), scanning electron microscopy (SEM), thermogravimetric analysis (TGA), Z-potential measurement, and XRD analyses.

##### 2.2.1.1 Scanning Electron Microscopy and Transmission Electron Microscopy

Morphological properties of AC were investigated by TEM. TEM micrographs were obtained using a Tecnai G2 S-TWIN microscope with adjustable voltage between 20 and 200 kV and it was also equipped with a high-resolution camera (Eagle 4K). SEM analyses were conducted using a Nova NanoSEM 450, which can provide information on the crystalline structure, surface topography, electrical behavior, and chemical composition.

##### 2.2.1.2 Thermal Gravimetric Analysis

TGA was used to establish both the thermal stability of the AC and then to check IPM weight loss at 310 °C caused by molecular degradation.^49^ Such an analysis was performed under N_2_, equilibrated at 100 °C, and then heated to 900 °C at a ramp of 10 °C/min by using a TA-Instrument (Q500, Milan, Italy).

##### 2.2.1.3 Zeta Potential Measurements and DLS

DLS and zeta potential measurements were carried out to obtain hydrodynamic diameter measurements, poly dispersive index, and potential Z of AC in an aqueous environment. The device used was a Zetasizer Nano-ZSP (Malvern, Worcestershire, UK) equipped with a helium–neon laser of 4 mW output power with a fixed wavelength of 633 nm (wavelength of laser red emission). The instrument software programmer calculated the zeta potential using the Henry equation and through electrophoretic mobility by applying a voltage of 200 mV. Two sets of samples were prepared, each containing seven aqueous solutions at different pH (from 2 to 14). To adjust the solution’s pH, HCl and NaOH solutions were used. In one of these sets, AC (1 mg/mL) was added to obtain a homogeneous dispersion. Experiments were carried out at a constant temperature (25.0 ± 0.1) °C. These DLS measurements were carried out in triplicate with the aim of verifying the reproducibility. Each measurement was constituted of 12 scans of 60 s to define the mean hydrodynamic radius and its error. The measurements were carried out at a temperature of 25 °C using a DTS 0012 standardized polystyrene cuvette for the analysis.

##### 2.2.1.4 Specific Surface Area and Porosimeter Analysis (BET)

Porosity and surface area are two important physical properties that define the effectiveness and quality of AC as an adsorbent. In this work, the surface area of the adsorbent was measured by using the Brunauer–Emmett–Teller (BET) technique, while the nonlocal density functional theory was used to determine the total pore volume. A small amount of the solid (ca. 0.02 g) was outgassed for around 15 h at 573 K under vacuum (*P* < 0.7 Pa). Then, the pore structure characteristic and specific surface area of the prepared samples of ACs were determined by nitrogen adsorption at 77 K by the surface area analyzer ASAP 2020 apparatus.

##### 2.2.1.5 XRD Analysis

The crystal structure of the AC was determined by obtaining X-ray powder diffractograms recorded using a Rigaku miniflex 600 X-ray diffractometer (Rigaku Co., Tokyo, Japan). The instrument was operated at 600 W using Cu Kα radiation; diffraction intensities were measured by a fixed time step scanning method in the range of 5–70° (2⊖).

##### 2.2.1.6 Powder Grain Size Distribution

The adsorbent particle size was directly measured using a laser light scattering instrument (Mastersizer 3000, Malvern Instruments Ltd., Malvern, U.K.) with an optic of 10 nm to 3.5 mm. The AC powder was dispersed by using the Aero S dry powder dispersion system.

##### 2.2.1.7 FTIR Analysis

Fourier transform infrared (FTIR) spectra were obtained using an FTIR 4700LE (JASCO, Tokyo, Japan) using attenuated total reflectance, and the spectrum was obtained at a resolution of 2 cm^–1^ over the range of 400–4000 cm^–1^. First, the sample of pristine PAC was mixed with potassium anhydrous bromide (KBr) (m/m, 1:2000) and the mixture was pressed as a pellet prior to analysis. KBr was used also as the reference material. To identify the interaction between IPM and AC Fluka, the pellet of AC and KBr mixture was covered with a drop of Isovue solution.

##### 2.2.1.8 UV–Vis Analysis

To obtain the IPM concentration, UV–vis spectrophotometry was conducted. Samples were diluted to obtain an absorbance value between 0.2 and 0.8 in agreement with the Lambert–Beer equation. A Varian Cary-50 spectrophotometer was used. A typical spectrum is shown in the Supporting Information (Figure S1). A calibration curve was recorded at 240 nm (Figure S2).

##### 2.2.1.9 HPLC Analysis

High-performance liquid chromatography (HPLC) analyses were carried out using an Agilent Infinity 1200 HPLC instrument. The HPLC method used employs a Zorbax SB-Phenyl (80 Å 5 μm, 250 × 4.6 mm, Agilent Technologies) column. Elution was performed using water as solvent A and acetonitrile as solvent B. The UV detector was set at λ = 240 nm, and the flow rate was set at 1 mL/min. Certified Reference Material IPM Pharmaceutical Secondary Standard was used as an external standard. The calibration curve with HPLC is reported in the Supporting Information (Figure S3).

#### 2.2.2 Preparation of the IPM Solution

ISOVUE-370 has a tendency to crystallize because of the high IPM concentration (755,000 mg/L). For this reason, 500 mL of ISOVUE-370 solution was warmed at 250 °C on a hot plate^49^ to dissolve the crystals and successively used to prepare 50 mL of aqueous solution (called stock solution) with an IPM concentration of 7550 mg/L. The latter was further diluted in a glass flask to obtain a final 1 L solution at the concentration of 100 mg/L. The obtained solution was sonicated using an ultrasonic bath (FALC LBS Instruments, Italy) and no pH adjustment was made.

#### 2.2.3 Kinetic and Thermodynamic Experiments

**Figure 1:**
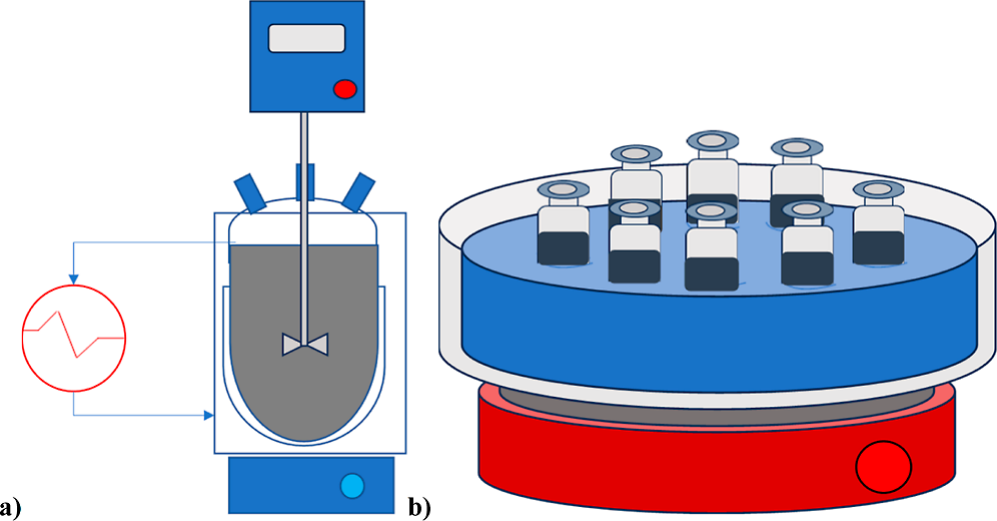
Scheme of the equipment adopted for the: (a) kinetic and (b) thermodynamic investigations.

The adsorption kinetics tests were carried out in a 1.5 L glass jacketed three-necked flask equipped with an impeller to provide the stirring of the system at the desired rotational speed of 800 rpm and a PTFE tube to collect samples of the solution over time by a syringe. 1 L of IPM solution was loaded into the reactor and heated at a fixed temperature using a thermostat connected to the flask’s jacket for the entire duration of the test. The sketch of the equipment is reported in Figure 1A.

To investigate the adsorption kinetics, several experiments were conducted by varying the adsorbent bulk density (ρ_bulk_), the IPM initial concentration (*C*_0_) and the temperature (*T*). The experimental conditions are given in Table 3. As revealed, the experimental matrix consists of 11 tests, where the adsorption kinetics was measured varying the above-mentioned experimental conditions. Three experiments were conducted by varying the temperature, three varying the IPM initial concentration, and seven experiments varying the sorbent loading. The latter effect was deeply investigated to recheck the thermodynamic information collected at the plateau observed at the end of each kinetic test.

**Table 3:** Experimental Conditions for the Kinetics Experiments^a^

test	*T* [K]	ρ_bulk_ [kg/m^3^]	*C*_0_ [mol/m^3^]
1	303	1.00	0.13
2	303	0.50	0.13
3	303	0.25	0.13
4	303	0.10	0.13
5	303	0.05	0.13
6	303	0.01	0.13
7	303	0.005	0.13
8	293	0.25	0.13
9	313	0.25	0.13
10	303	0.25	0.09
11	303	0.25	0.16

a The stirring rate was fixed at *v* = 800 rpm for all the experiments.

Samples were withdrawn periodically and analyzed via UV–vis and HPLC methods to ensure the correct quantification of the pollutant and verify eventual side products.

PAC regeneration experiments were conducted at different pH using saturated PAC with IPM. The saturated PAC samples were prepared putting in contact 2 g of PAC with a solution of IPM of 2000 ppm. The suspension was well stirred and monitored until the IPM concentration in the liquid phase did not change. The solid was filtered and put in an oven at 60 °C for 2 h for drying purposes. In this way, an aliquot of 0.1 g of saturated PAC was placed in contact with 10 mL of ultrapure water, adjusting the pH with either HCl or NaOH 0.1 M solutions (pH 2, 7, and 9), to identify the optimal pH to regenerate the sorbent. Finally, PAC stability was investigated conducting XRD analyses on both unused PAC and saturated PAC. The desorption percentage was defined as % desorption = 100 × *q*_IPM_/*q*_e_, with *q*_e_ the saturation uptake and *q*_IPM_ the amount of released IPM per mass of sorbent calculated using the concentration measured after the regeneration experiment (*C*_B_) reported per sorbent loading, as *q*_IPM_ = *C*_B_/ρ_bulk_.

To study the adsorption thermodynamics of IPM onto activated charcoal, 10 mL of the solution (initial concentration 100 mg/L) was mixed with different amounts of activated charcoal in seven different glass vials. The sketch of the equipment is reported in Figure 1B. A magnetic stir bar was introduced, the vials were closed, labeled, and placed in a water bath for 24 h. The tests were conducted at three different temperatures: 293, 303, and 313 K. All the experiments were conducted three times to reduce variability and uncertainty. The error bars reported in each figure were calculated with the average standard deviation obtained from repeated experiments (5%).

After each experiment, the samples were centrifugated for 60 min to allow the deposition of the solid on the bottom of the vials. The liquid phase was recovered through microfiltration and analyzed by both UV spectrophotometry and HPLC to detect the concentration of the solute.

The amount of IPM adsorption at equilibrium *q*_e_ (mg/g) was calculated using eq 1.
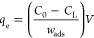
1where *C*_0_ and *C*_L_ (mg/L) are the IPM initial concentration and the concentration of the solute at the equilibrium, respectively, *V* (L) is the volume of the solution, and *w*_ads_ (g) is the mass of adsorbent used.^50^

#### 2.2.4 Modeling and Numerical Strategies

The interpretation of the kinetic data was performed with the adsorption dynamic intraparticle model (ADIM) developed by Russo et al.^51^ This model was used to give deeper insights into the pore diffusion and surface diffusion phenomena occurring in the described system. The latter is conceptually divided into three domains: a bulk liquid phase in which the solute concentration is assumed constant (at a fixed time *t*) up to the liquid film around the particle, where external diffusion takes place; a liquid phase and a solid phase inside the particle between which a local equilibrium is assumed (expressed in terms of the adopted equilibrium isotherm) and where the solute concentration changes with the time along the particle radius. In addition, by considering an isothermal system and by assuming monomodal particle sizes, it is possible to derive for the batch system the mass balance using eqs 2 and 3.
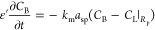
2
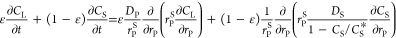
3

Equation 2 takes into account the fluid–solid diffusion limitations, while in eq 3 the sum of the accumulation terms for both the solid and liquid phases is equal to sum of the pore and the surface intraparticle diffusion limitations. A set of boundary conditions is necessary to solve this system of partial differential equations. eqs 4 and 5 represent the symmetry condition at the center of the particle (*r*_p_ = 0) for both the adsorbed phase and liquid and eq 6 defines the surface steady-state hypothesis.
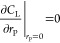
4
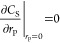
5
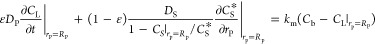
6

The local equilibrium condition inside the particle is expressed by the Langmuir adsorption isotherm (eq 7), which is needed to evaluate the solute concentration in the solid.
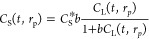
7

Furthermore, the determination of chemical and physical parameters like the pore diffusivity *D*_P_, the external mass transfer coefficient *k*_m_, and the surface diffusivity *D*_S_ is essential.

The pore diffusivity can be calculated from the following expression (eq 8)
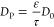
8where *D*_0_ is the molecular diffusivity, estimated using Wilke–Chang correlation^52^ (eq 9), ε is the porosity of the solid, and τ is the tortuosity factor.
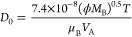
9*k*_m_ was fixed to a large value since we assumed the external mass transfer limitations to be negligible under the adopted experimental conditions. On the other hand, the value of the surface diffusivity *D*_S_, which depends on the molecular interaction between the adsorbate and the adsorbent, cannot be calculated from any mathematical expression. Thus, it must be fitted on the experimental data.

The simultaneous numerical solution of this system of ordinary differential equations, partial differential equations, and algebraic equations was made by using an algorithm based on the method of lines and provided by the software gPROMS Model Builder. A second order centered finite difference method of approximation was used as the solver, discretizing the particle in 100 collocation points.

The thermodynamic parameters, namely, the Gibbs free energy and the enthalpy and entropy values (respectively, Δ*G*°, Δ*H*°, and Δ*S*°) were calculated at different temperatures (i.e., *T* = 293, 303, and 313 K). The thermodynamic parameters were computed adopting eqs 10 and 11.^53^
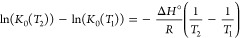
10
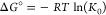
11where *K*_0_ is the ratio between the IPM concentration adsorbed on PAC and at the equilibrium in the liquid phase (i.e., *K*_0_ = *C*_S_/*C*_L_), *R* is the ideal gas constant (8.314 J mol^–1^ K^–1^), *T* is the absolute temperature (K), and *T*_1_ and *T*_2_ are two different temperatures.

## 3 Results and Discussion

### 3.1 Adsorbent Characterization

**Figure 2:**
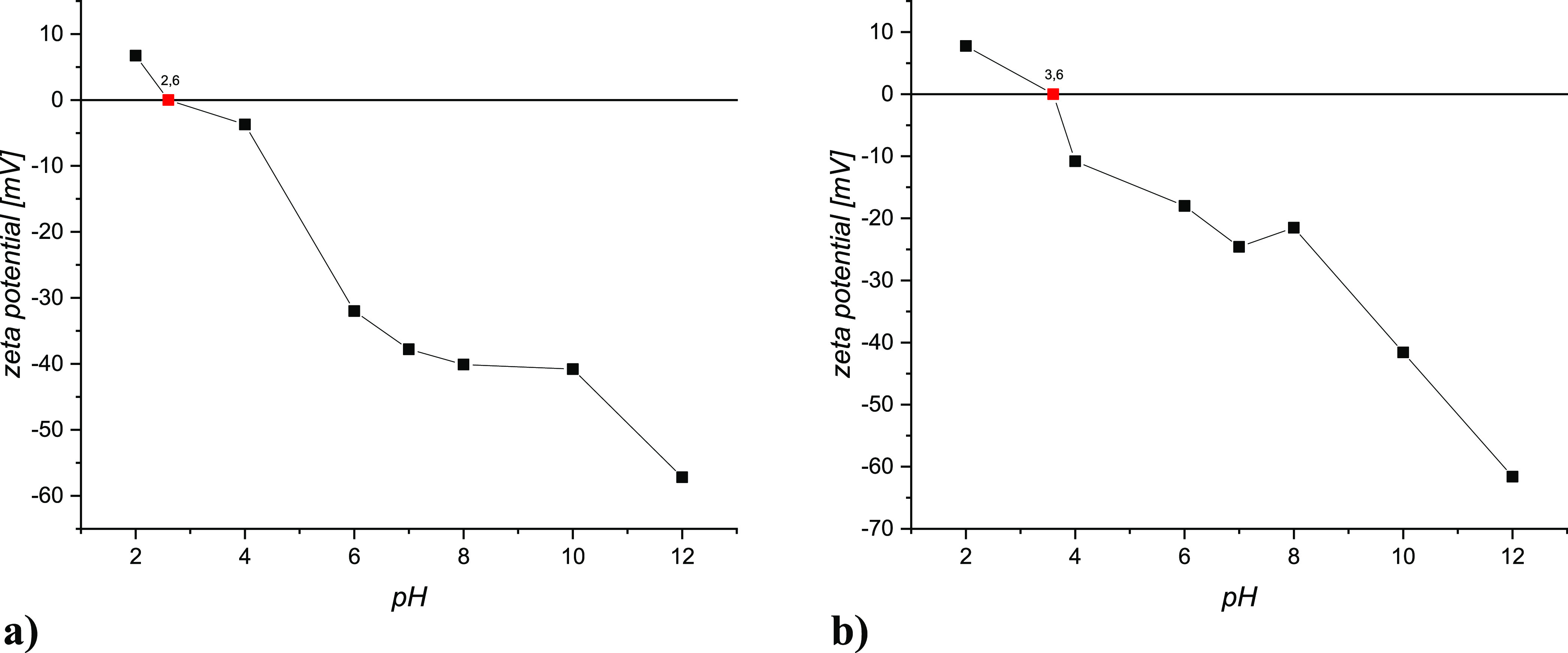
(a) Zeta-potential micrographs of AC Fluka at different pH alone and (b) in contact with IPM.

The value of pHpzc of AC was determined by the influence of all the functional groups, i.e., the pH at which the sum of the total surface charges on carbon were zero. At pH < pHpzc, the carbon surface has a net positive charge, while at pH > pHpzc the surface has a net negative charge. The types of functional groups present on the surface and pHpzc are important characteristics for any AC as they indicate: type of AC (either H- or L-type), the acidity/basicity of the adsorbent, and the net surface charge of the carbon in solution. The most characteristic acid functional groups present on the AC are carboxylic, phenolic, and lactonic.^54^ The functional groups with basic properties are oxygen-containing species such as pyronic, ketonic, chromenic, and p-electron system of carbon basal planes.^55^ Z-potential measurements of solution at different pH revealed that the pHpzc value of Fluka AC was 2.7, while in the presence of IPM solution, pHpzc shifts at 3.6 (see Figure 2). Mestre et al.^17^ observed that IPM is a neutral molecule in aqueous solutions (at pH = 5), thus π–π interactions can take part in the adsorption on a slightly negative charged surface. Thus, under acidic conditions, both AC and IPM are positively charged.

**Figure 3:**
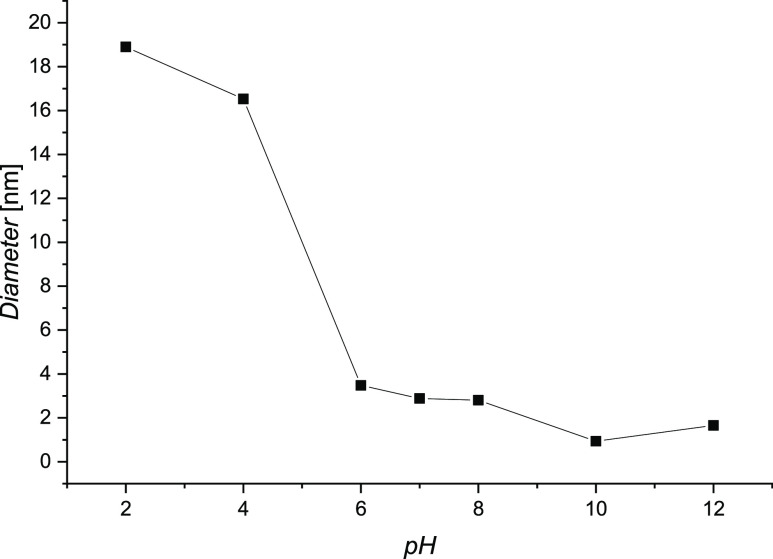
DLS results of IPM and the AC solution at different pH values.

DLS results reveal aggregate formation at pH = 2.4 created by IPM and AC interaction in aqueous solution (Figure 3).

Specific surface area (SSA) was determined from the linear part of the BET equation (BET SSA). The BET surface area was found to be 1875 m^2^/g and the total pore volume was 1.25 cm^3^/g. While the volume in pores is 0.018 cm^3^/g, the total volume in pores is 1.16 cm^3^/g and the total area in pores is 1754 m^2^/g. The results are in line with those reported in the literature.^56^

**Figure 4:**
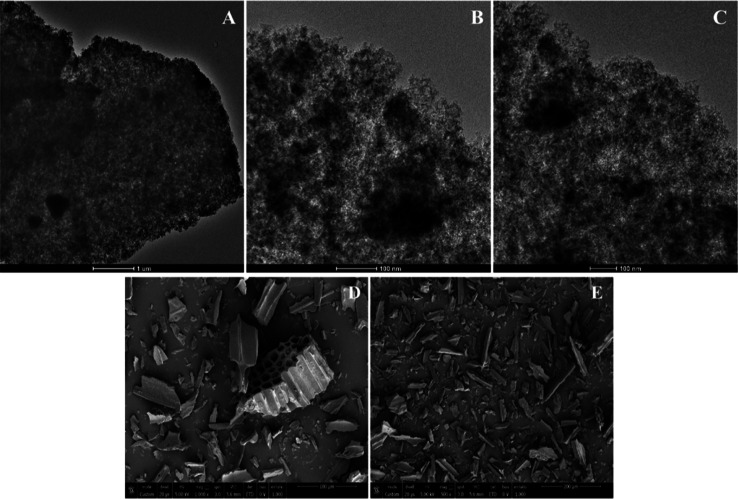
TEM (A–C) and SEM micrographs (D,E) of the AC Fluka.

The SEM micrographs were conducted to investigate the morphology of the sorbent used (Figure 4). The micrographs show lamellar composition of the AC Fluka.^57^ The TEM microphotographs reveal the presence of disordered and curled single carbon layers.

**Figure 5:**
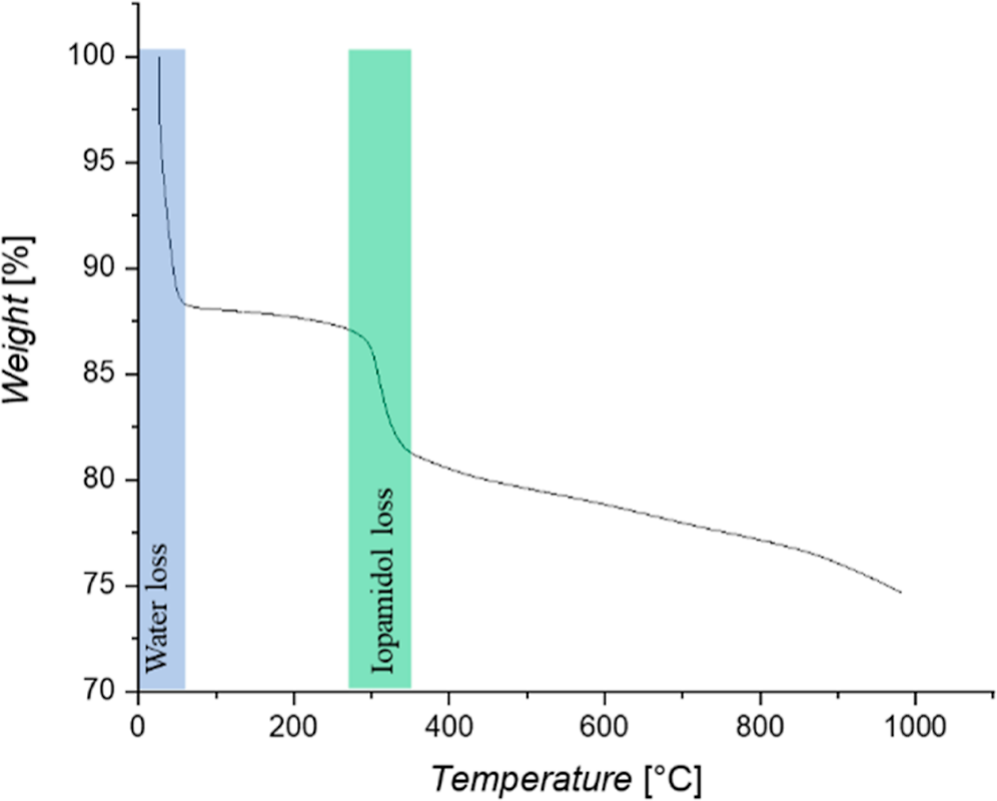
TGA analysis of PAC Fluka after the IPM adsorption test.

TGA results are reported in Figure 5. The TGA analyses performed on the PAC samples after the adsorption test with IPM underline that significant weight loss at 300 °C was observed upon IPM adsorption (8%), as reported in Figure 5. This proves that IPM was effectively adsorbed onto CPAC from the aqueous solution.

### 3.2 Adsorption Thermodynamics

**Figure 6:**
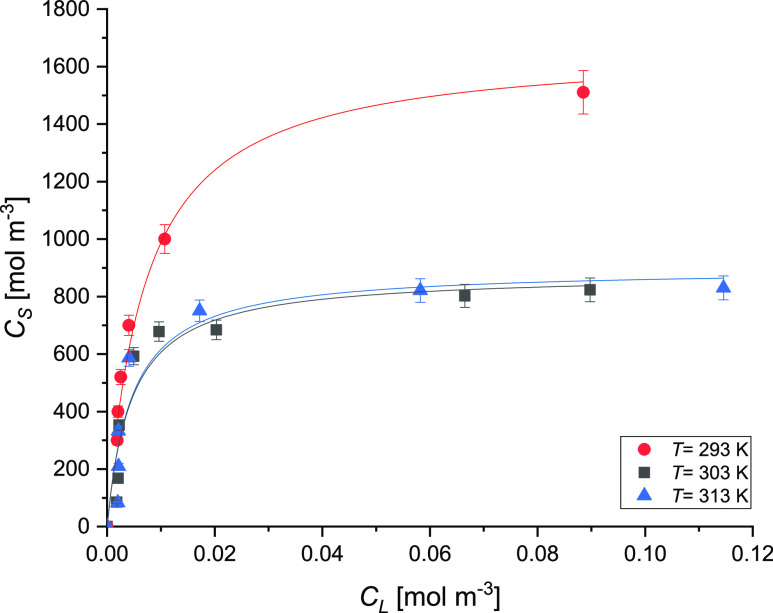
Adsorption isotherms at different temperatures (*T* = 293, 303, and 313 K). The symbols represent the experimental data, whereas the solid lines represent the fitting with the Langmuir isotherm.

To describe how the adsorbate molecules are distributed between the liquid and solid phases when the process of adsorption reaches an equilibrium state, the adsorption equilibrium is obtained. The trend of the adsorption isotherms of IPM is shown in Figure 6.

The obtained results suggested that the adsorption of IPM onto AC is an exothermic process since the solute uptake is higher at lower temperatures. Furthermore, there is no appreciable effect of the temperature between the results obtained at 303 and 313 K.

Several models (i.e., Langmuir, Freundlich, and Sips) were applied to describe the collected data. The results in terms of statistical analysis and fitted parameters are reported in Table 4.

**Table 4:** Estimated Values of Langmuir, Freundlich, and Sips Adsorption Parameters at Different Temperatures

adsorption isotherm model	*T* [K]	293	303	313	*R*^2^	*R*^2^_adjusted_
Langmuir	*C*_s_^*^ [mol m^–^^3^]	1700 ± 200	800 ± 40	800 ± 40	0.92	0.90
	*b* [m^3^ mol^–^^3^]	140 ± 30	200 ± 50	200 ± 50		
Freundlich	*K*_F_ [(mol m^–^^3^)^1^^–^^n^]	3200 ± 500	1600 ± 400	1600 ± 400	0.84	0.78
	*n* [-]	3.3 ± 0.4	4 ± 1	4 ± 1		
Sips	*C*_s_^*^ [mol m^–^^3^]	1600 ± 100	760 ± 40	800 ± 50	0.97	0.95
	*b* [m^3^ mol^–^^3^]	170 ± 80	330 ± 100	340 ± 100		
	*n* [-]	1.0 ± 0.2	0.4 ± 0.3	0.3 ± 0.2		

The Freundlich model is inadequate to describe the collected data as revealed by the lowest *R*^2^ value and the high errors on the fitted parameters. By comparing Langmuir and Sips isotherms, the Sips model allowed to achieve the lowest adjusted *R*^2^ (i.e., *R*^2^ adjusted by the number of fitting parameters, *R*^2^_adjusted_) but the related parameters are characterized by larger confidence intervals compared with the Langmuir model. Thus, it is possible to conclude that the adsorption occurs as a monolayer process on energetically and homogeneous equivalent sites.

The collected information was very useful to retrieve the thermodynamic parameters reported in Table 5, which were calculated using the Langmuir model parameter values reported in Table 4.

**Table 5:** Thermodynamic Parameters Obtained for IPM Adsorption on PAC

*T* [K]	Δ*H*° [kJ/mol]	Δ*S*° [kJ/mol]	Δ*G*° [kJ/mol]
293	–27.39	–0.024	–34.6
303	–27.39	–0.027	–35.8
313	–27.39	–0.028	–36.1

The negative values of Δ*G*° indicate that the adsorption process is spontaneous and IPM molecules have high affinity to PAC. The negative value of Δ*H*° indicates that the adsorption process is relatively exothermic. In detail, the adsorption enthalpy value was found to be equal to Δ*H*° = −27 kJ mol^–1^, confirming the values reported in the literature.^48^ The slightly negative Δ*S*° value indicates that when IPM molecules are adsorbed on the PAC surface, it is possible to obtain a slightly ordered structure.

### 3.3 PAC Stability, Regeneration, and IPM–PAC Interaction Mechanism

**Figure 7:**
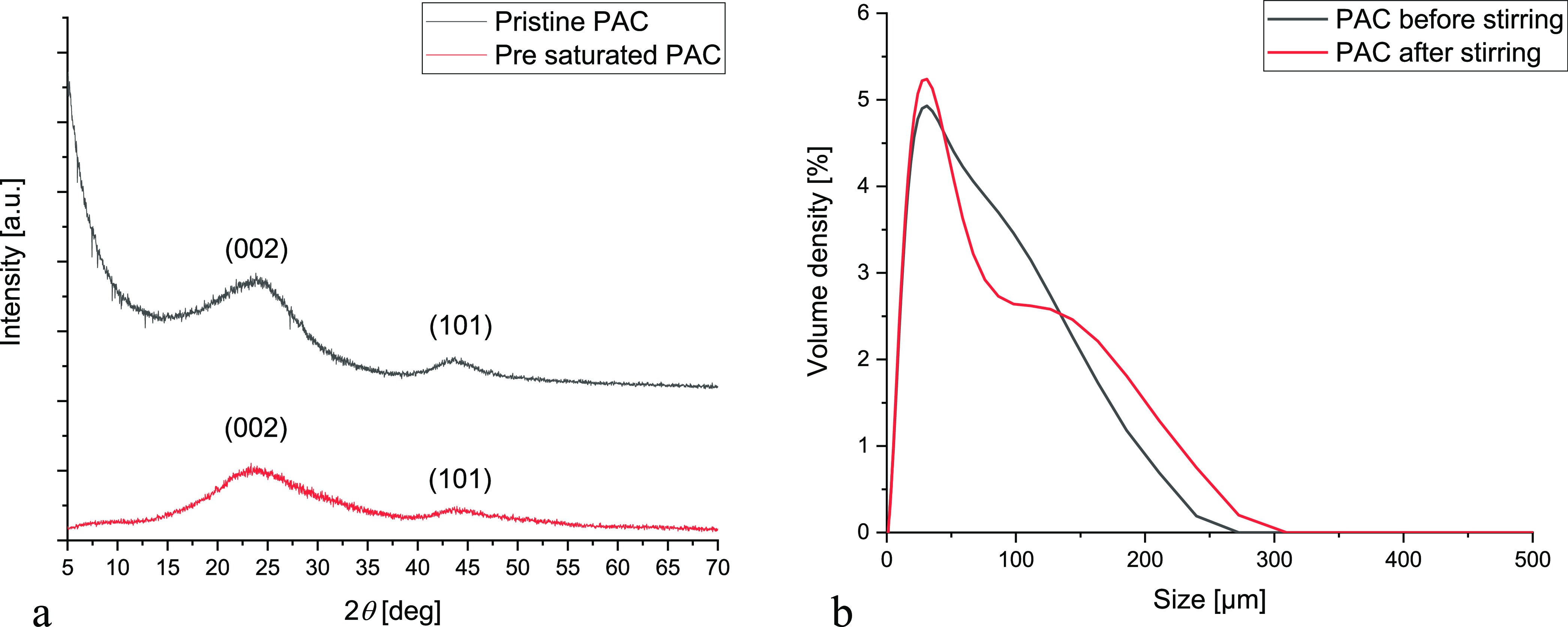
(a) XRD analyses on both PAC and presaturated PAC with IPM. (b) Particle size distributions measured for pristine PAC dispersion (1 g/L) and PAC dispersion after stirring (*v* = 800 rpm, *t* = 3 h).

XRD analyses were conducted on both PAC and PAC presaturated with IPM (see Methods for details). The results reported in Figure 7a clearly show the amorphous nature of the sorbent, both prior and post saturation with IPM. Both samples show two diffraction peaks at 2θ = 25° and 44.5°, corresponding to, respectively, the planes (002) and (101), indicating a graphitic hexagonal structure.^58^

The sorbent was demonstrated to be stable, as revealed from the experimental XRD patterns reported in Figure 7a, where it is evident that the XRD patterns are very similar; thus, it is possible to exclude any change in PAC structure.

Further, the PAC mechanical stability was investigated. Pristine PAC dispersion in water (0.1 g/L) was put under vigorous stirring (*v* = 800 rpm) for 3 h. The sorbent size distributions were measured both prior to and after stirring, clearly indicating only small differences (see Figure 7b).

**Figure 8:**
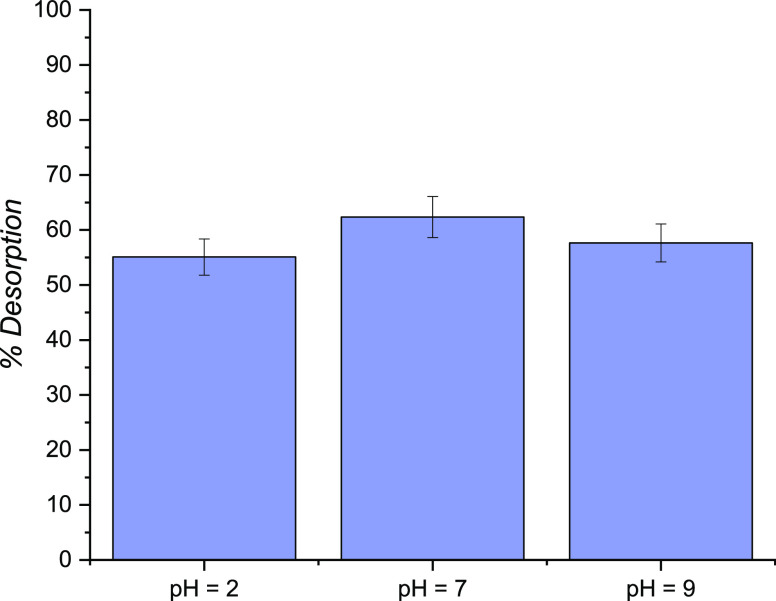
Investigation of saturated PAC regeneration. Experimental conditions are *C*_0,IPM_ = 0 mol/m^3^, *T* = 303 K, and *v* = 800 rpm.

Regeneration experiments were conducted, placing in contact presaturated PAC with ultrapure water at a chosen pH (2, 7, and 9). The results are reported in Figure 8.

As revealed, the desorption percentage is almost constant, varying the pH from 2 to 9, indicating a complex release mechanism that requires further investigation.

**Figure 9:**
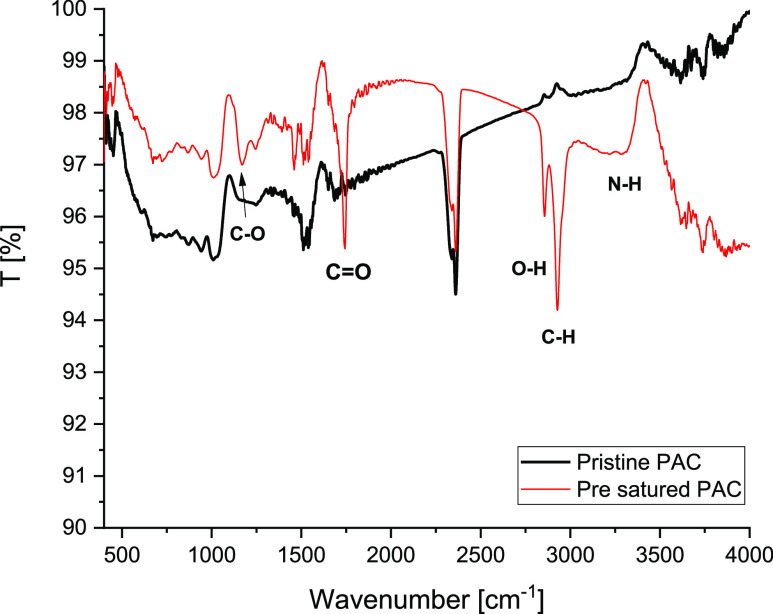
FTIR analyses of both pristine and presaturated PAC.

The interaction between IPM and PAC was investigated via FTIR spectroscopy, measuring both the transmittance of pristine PAC and presaturated PAC with IPM (see Figure 9).

The FTIR spectrum of the presaturated PAC shows different adsorption bands compared with the pristine PAC, a band at 1600–1800 cm^–1^, corresponding to C=O stretching, a band at 1050 and 3300 cm^–1^, assigned to, respectively, the C–O and O–H/N–H stretching modes of IPM molecules. The presence of the new bands indicates that the mentioned functional groups are strongly perturbed when interacting with IPM.^48^

### 3.4 Kinetic Experimental Results

**Figure 10:**
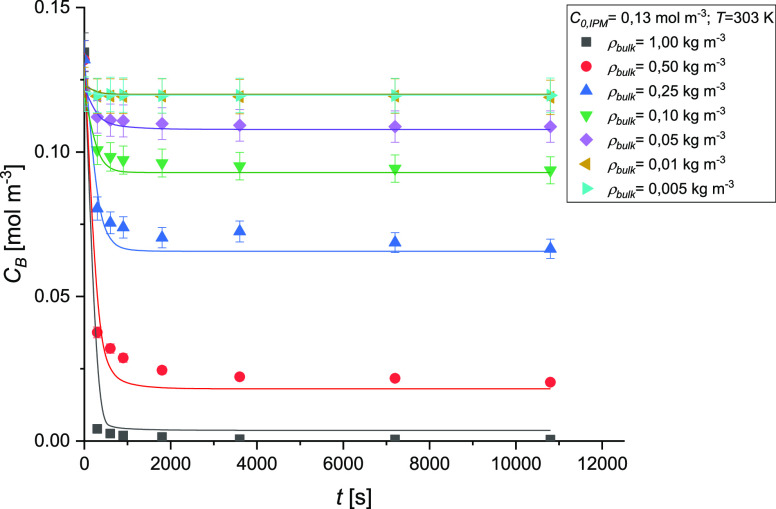
Effect of the adsorbent bulk density on the adsorption kinetics of IPM on AC. Experimental conditions are *C*_0,IPM_ = 0.13 mol/m^3^, *T* = 303 K, and *v* = 800 rpm.

The effect of sorbent load on the adsorption rate of the IPM was investigated at *T* = 303 K, *v* = 800 rpm, and by fixing the initial concentration of IPM at *C*_B,0_ = 0.13 mol m^–3^. Figure 10 shows the trend of the IPM bulk concentration at different adsorbent bulk densities. It can be noticed that by increasing the sorbent bulk density, an increase in the adsorption capacity is obtained. This is true from the bulk density value of 0.05 kg m^–3^ since there is no effect on the IPM uptake between the experiments performed with 0.005 and 0.01 kg m^–3^ of adsorbent. Increasing the sorbent dosage at a fixed IPM initial concentration provided more available adsorption sites for IPM and thus increased the extent of removal of IPM from the solution. A typical UV–vis spectrum of IPM before and after contact with AC was reported (Figure S4).

**Figure 11:**
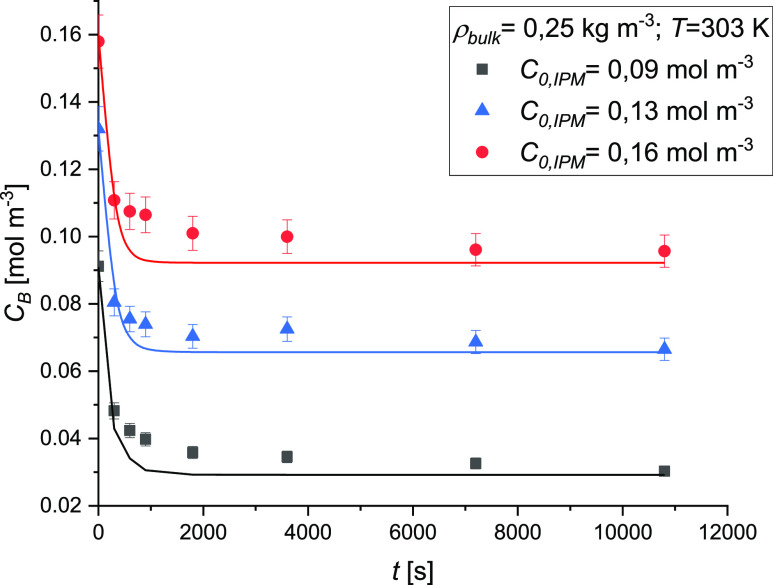
Effect of the initial concentration of IPM on the adsorption kinetics, experimental conditions: ρ_bulk_ = 0.25 kg m^–3^, *T* = 303 K, and *v* = 800 rpm.

Further experiments were conducted to test the effect of the initial concentration of IPM in solution at a temperature of 303 K and a bulk density value of 0.25 kg m^–3^. Three different initial IPM concentrations were tested: 0.09, 0.13, and 0.16 mol m^–3^. As reported in Figure 11 with the initial concentration variation of the solute, the adsorption capacity of AC did not change; in fact, the amount of drug removed was 0.06 mol m^–3^. This observation is in line with the Langmuir model, determined in the previous section, as the sorbent monolayer is saturated in the adopted concentration range. Thus, we can conclude that the Langmuir model is more adequate to describe the thermodynamic data than both Sips and Freundlich models.

**Figure 12:**
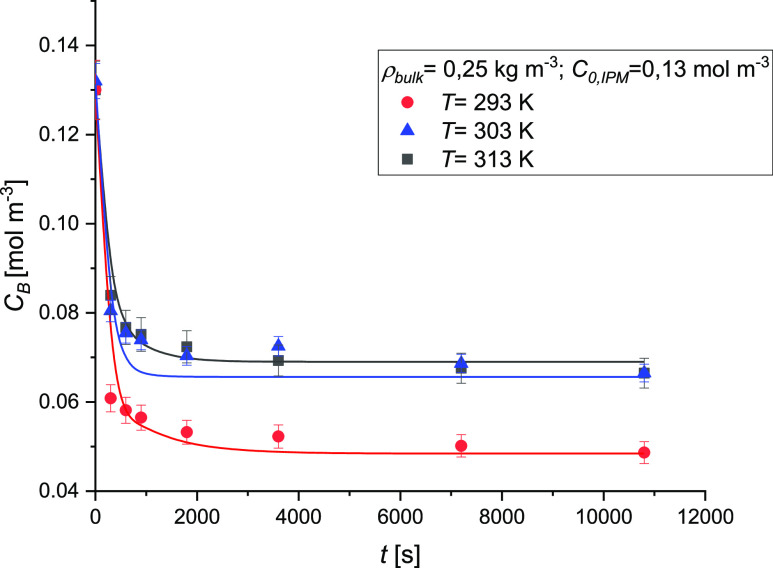
Effect of the temperature on the adsorption kinetics of IPM on AC. Experimental conditions are *C*_0,IPM_ = 0.13 mol/m^3^, ρ_bulk_ = 0.25 kg m^–3^, and *v* = 800 rpm.

The effect of the temperature on the adsorption kinetics is shown in Figure 12. As the temperature increases, a decrease in the IPM uptake is obtained, confirming the exothermic nature of the process as already reported in the previous paragraph. In fact, the IPM uptake is 33% higher at 293 K compared to that obtained at 303 and 313 K.

Additionally, the values of the surface diffusivity *D*_s_ were estimated at the different temperatures (Table 6). According to Russo^50^ et al., *D*_s_ follow an Arrhenius-like trend, expressed by the mathematical equation reported in eq 12.
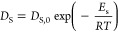
12

**Table 6:** Estimated Values of *D*_s_ at Different Temperatures

*T* [K]	*D*_s_ × 10^12^ [m^2^ s^–^^1^]
303	8 ± 2
313	9 ± 1
323	9.9 ± 0.1

**Figure 13:**
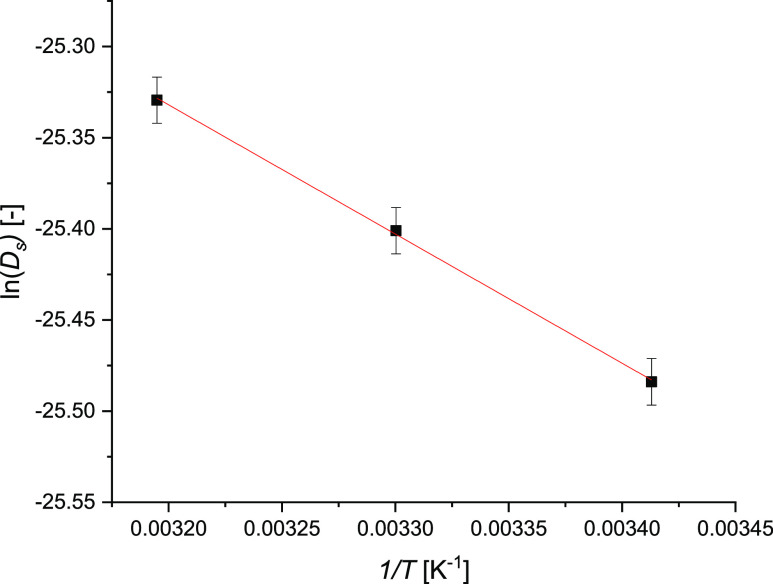
Linearized trend of the surface diffusivity *D*_s_ vs *T*.

By plotting the natural logarithm of *D*_s_ as a function of 1/*T*, a linear trend is obtained (Figure 13). The value of the surface diffusion activation energy *E*_s_ was calculated from the slope of the fitting line, and it was found to be equal to 6 ± 1 kJ mol^–1^.

**Figure 14:**
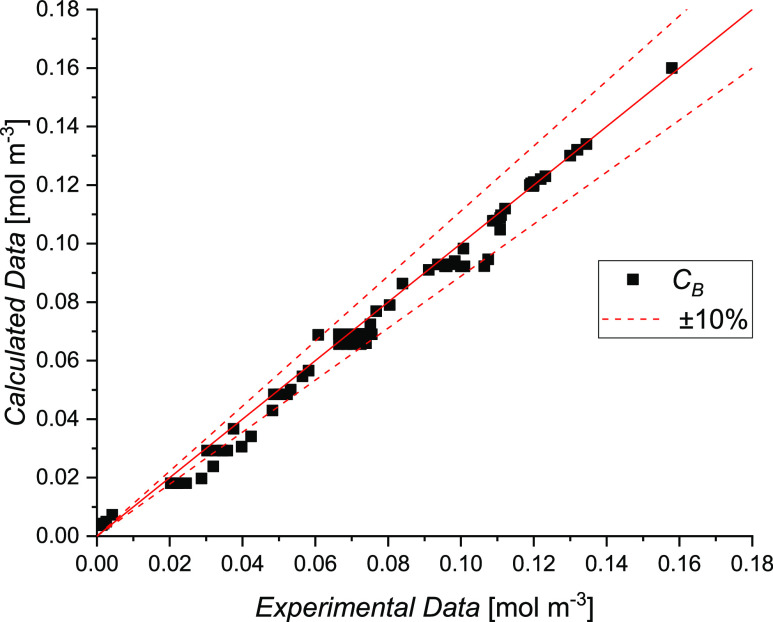
Parity plot of IPM bulk concentration, including all the collected data of the kinetic experiments.

The parity plot for all of the kinetic and thermodynamic data fitting is shown in Figure 14. Comparing the experimental results and model predictions, all the points fall within a confidence interval of ±10%. In conclusion, the overall goodness of the fit is corroborated by the coefficient of determination *R*^2^ that equals 0.99.

## 4 Conclusions

In this work, the adsorption of IPM on powdered AC was studied. The adsorbent characterization confirmed the high surface development and porosity typical of AC. It has been seen that the zeta potential value of the adsorbent when placed in contact with the IPM increases by one unit (from 2.6 to 3.6). DLS investigations showed the formation of aggregates between carbon and IPM at pH < 6.

The thermodynamics experiments were carried out in batch mode, finding that IPM adsorption on activate carbon is exothermic (Δ*H*° = −27 kJ mol^–1^), as under the same experimental conditions, IPM is removed 33% more at *T* = 293 K compared to *T* = 303 and *T* = 313 K. From the data analysis, the Langmuir isotherm was found to be the most appropriate in describing the thermodynamic data compared with the performance obtained using both Freundlich and Sips models. This finding was further demonstrated within the description of the kinetic experiments conducted at different initial IPM concentrations.

The kinetic experiments were also conducted in batch mode. It was revealed that the adsorbent loading must be at least 0.05 kg m^–3^ to show a good removal capacity of the pollutant. The increase of the initial concentration of IPM in the solution, at fixed sorbent bulk density, does not affect the removal capacity of AC, further demonstrating the existence of a saturated monolayer under the adopted experimental conditions. The ADIM model was implemented and adopted in describing the kinetic data, always obtaining a good fit of all the collected data. The model provided insights into the diffusion mechanism of IPM through the determination of the surface diffusivity values, *D*_s_, and consequently the surface diffusion activation energy value, *E*_s_ = 6 ± 1 kJ mol^–1^.

In perspective, the collected information will be used to design an adsorption column working in continuous mode, allowing the process scale-up from batch to continuous operation, which is surely useful for industrial applications.

## Supporting Information Available

UV–vis absorption spectra of IPM solutions, UV–vis calibration curve, HPLC calibration curve, and UV–vis spectra of IPM before and after contact with AC Fluka (PDF)

## List of symbols

*a*_sp_: geometrical specific surface area (m^2^/m^3^)
*b*: Langmuir adsorption constant (m_liq,P_^3^/mol)
*C*_0_: adsorbate initial concentration (mol/m^3^)
*C*_B_: solute bulk concentration (mol/m^3^_BULK_)
*C*_L_: solute concentration in the liquid of the pores (mol/m_liq,P_^3^)
*C*_s_: solute concentration in the solid (mol/m^3^_sol,P_)
*C*_s_^*^: saturation solute solid concentration (mol/m_sol,P_^3^)
*D*_0_: molecular diffusivity (m^2^/s)
*D*_p_: pore diffusivity based on the cross-sectional area (m^2^/s)
*D*_s_: superficial diffusivity (m^2^/s)
*D*_s0_: pre-exponential (m^2^/s)
*E*_s_: surface diffusion activation energy (kJ/mol)
*K*_0_: *C*_S_/*C*_L_ (−)
*k*_m_: mass transfer coefficient (m/s)
*q*_IPM_: concentration of IPM in solution (mg/g)
*q*_e_: amount adsorbed (mg/g)
*R*: ideal gas constant (J K/mol)
*r*_p_: particle radial direction (m)
*R*_p_: particle radius (m)
*t*: time (s)
*T*: temperature (K)
*V*: liquid volume (L)
*W*_ads_: adsorbent mass (kg/m^3^)

## Greek symbols

Δ*G*°: Gibbs free energy change [J/mol]
Δ*H*°: enthalpy change [J/mol]
Δ*S*°: entropy change [J/(mol K)]
ρ_bulk_: solid bulk density (kg/m^3^)
ε: particle porosity (m_liq,P_^3^/m_p_^3^)
τ: tortuosity factor

## Abbreviations

AC: activated carbon
CP, VP, NS: commercial carbons
CPAC: coconut powder AC
GAC: granular activated carbon
ICMs: iodinated X-ray contrast media
I-DBPs: detrimental iodinated byproducts
IPM: iopamidol
I-THMs: iodinated trihalomethanes
PAC: powdered Activated Carbon
PGAC: peat granular AC
PPAC: peat powder AC
PPCPs: personal care products
WPAC: wood powder AC
WWTPs: wastewater treatment plants
