# Supplementary material for: Iopamidol Abatement from Waters: A Rigorous Approach to Determine Physicochemical Parameters Needed to Scale Up from Batch to Continuous Operation
Source: Langmuir. 2023 Dec 12;39(51):18983–94. doi: 10.1021/acs.langmuir.3c02992 (PMC10753885; doi:10.1021/acs.langmuir.3c02992)
Supplement: Supplementary file 1 — la3c02992_si_001.pdf [file la3c02992_si_001.pdf]

Iopamidol abatement from waters: a rigorous approach to determine physico-chemical parameters needed to scale-up from batch to continuous operation

Rosanna Paparo[1,2], Michele Emanuele Fortunato[1], Gianfranco Carotenuto[3], Fulvio Uggeri[4], Luigi Nicolais[5], Martino Di Serio[1,2], Marco Trifuoggi[1,2], Vincenzo Russo[1*]

[1] University of Naples Federico II, Chemical Sciences Department, IT-80126 Naples, Italy
[2] CeSMA—Centre of Meteorologic and Avanced Thecnology Services, University of Naples Federico II, corso N. Protopisani 70, 80146 Naples, Italy
[3] Institute for Polymers, Composites, and Biomaterials, National Research Council, SS Napoli/Portici, Piazzale Enrico Fermi 1, 80055 Portici, Italy
[4] Bracco SpA, via Caduti di Marcinelle, 13, 20134, Milano, Italy
[5] Materias Srl, corso N. Protopisani 70, 80146 Naples, Italy

* v.russo@unina.it

Supporting Information

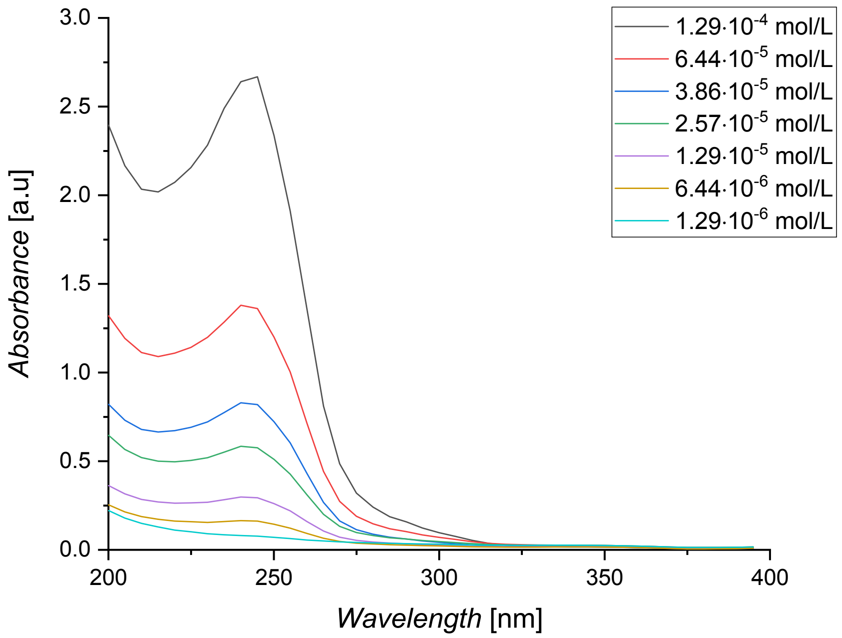

Figure S.1- UV-Vis spectra of iopamidol standard solutions.

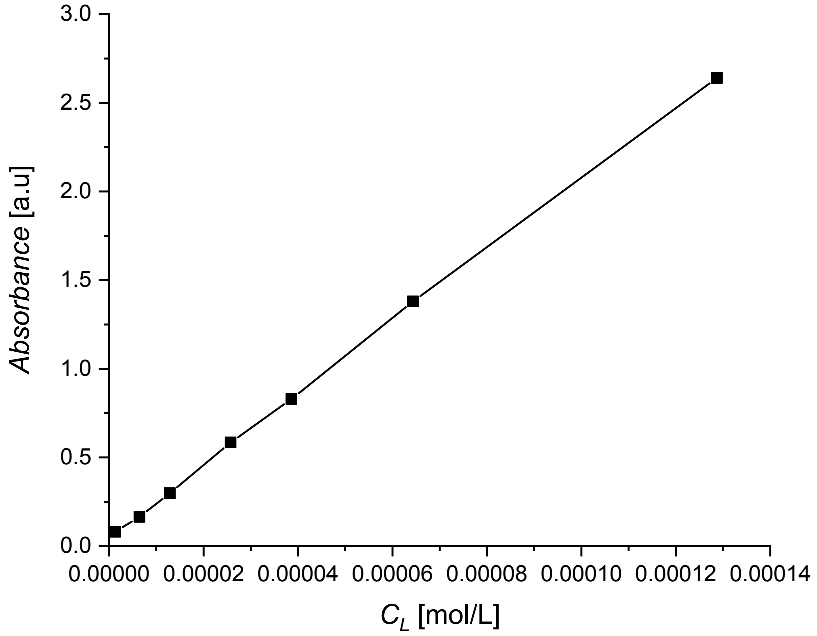

Figure S.2- UV-Vis calibration curve of iopamidol (IPM).

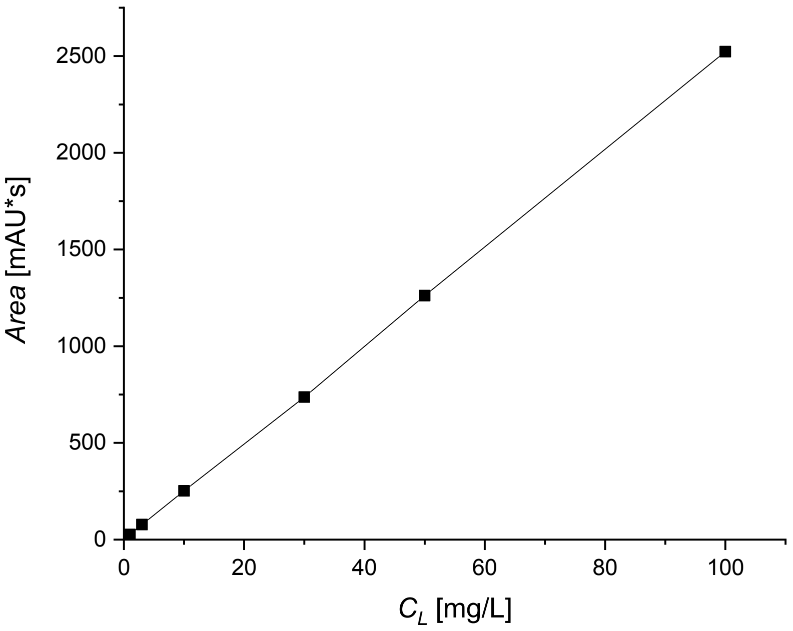

Figure S.3- HPLC calibration curve obtained for six standard solutions (1, 3, 10, 30, 50, 100 mg/L)

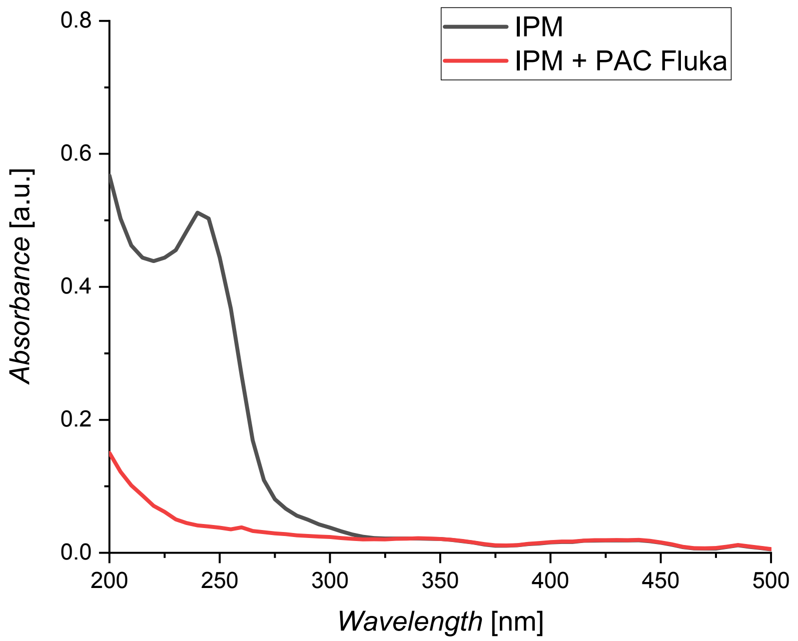

Figure S.4- UV-Vis spectra of iopamidol (IPM) before and after contact with activated carbon Fluka.
